# Supplementary material for: Noncovalent synthesis of homo and hetero-architectures of supramolecular polymers via secondary nucleation
Source: Nat Commun. 2024 Apr 30;15:3672. doi: 10.1038/s41467-024-47874-5 (PMC11063220; doi:10.1038/s41467-024-47874-5)
Supplement: Supplementary file 1 — Supplementary Information [file 41467_2024_47874_MOESM1_ESM.pdf]

Supplementary Information

Noncovalent synthesis of homo and hetero-architectures of supramolecular polymers via secondary nucleation

Srinu Kotha,[1] Rahul Sahu,[2] Aditya Chandrakant Yadav,[1,3] Preeti Sharma,[4] B. V. V. S. Pavan Kumar,[4] Sandeep K. Reddy,[2*] Kotagiri Venkata Rao[1*]

[1]Department of Chemistry, Indian Institute of Technology Hyderabad, Kandi, Sangareddy, Telangana-502284, India, E-mail: kvrao@chy.iith.ac.in

[2]Centre for Computational and Data Science, Indian Institute of Technology Kharagpur, West Bengal-721302, India, E-mail: skreddy@iitkgp.ac.in

[3]Department of Materials Science and Metallurgical Engineering, Indian Institute of Technology Hyderabad, Kandi, Sangareddy, Telangana-502284, India.

[4]Department of Chemistry, Indian Institute of Technology, Roorkee-247667, Uttarakhand, India.

Table of contents

**1. Supplementary Notes (Supplementary Note 1: General, Supplementary Note 2: Computational details and Supplementary Note 3: Mathematical Modelling and Fit Details)**	Page No. 2 to 6
**2. Supplementary Figures**	Page No. 7 to 33
**3. Supplementary Tables**	Page No. 34 to 35
**4. Supplementary References**	Page No. 36

1. Supplementary Notes

Supplementary Note 1: General

Electronic absorption spectra were recorded using a JASCO model V-770 UV-VIS-NIR spectrophotometer in a screw-capped quartz cell of 1 mm or 10 mm optical path length. Fluorescence spectra were recorded using a JASCO model FP-8300 spectrometer in a screw-capped quartz cell of 1 mm or 10 mm optical path length. SEM measurements were performed on a JEOL JIB4700F (FIB-SEM), where all samples were deposited on a pre-cleaned n-doped Si substrate by Spin coating and dried under air followed by vacuum at room temperature before the measurement. TEM measurements were performed on a JEOL JEM2100 (TEM), where the sample was deposited on a 300 mesh copper grid with lacey carbon support. Thin film XRD measurements were carried out on a Bruker Discover D8 X-ray diffractometer with Cu-Kα as the source. Optical microscopy experiments were performed on an Olympus IX83 inverted fluorescence microscopy, and the solution was deposited in 200 μm transparent rectangular capillaries.

Supplementary Note 2: Computational Details

To systematically explore the conformational space of the **2EH-PDI** molecule, a 2D potential energy scan was performed by varying the two imide position dihedral angles (C-N-C-C) using GAUSSIAN16 quantum chemical package, resulting in the generation of 121 conformations.[1] Next, each of the 121 structures was optimized using the PM6 method followed by the wB97XD/6-31G(d) method.[2-4] Using RMSD and visual inspection, we then discard identical conformations. This results in a total of six conformations, consisting of three chair-type conformations and three boat-type conformations and the energies are within ~ 5 kJ/mol. We computed the Boltzmann weighted factors of these six stable conformations at 363 K according to the formula:

$$pi = \frac{exp(-Ei/kT)}{\sum_1^6 \ exp(-Ei/kT)} \tag{S1}$$

Where E_i is the ground state energy of the conformation i and T = 363K.

The VMD software was utilized for visualization and making the Figures.[5]

Supplementary Note 3: Mathematical Modelling and Fit Details

The growth kinetics of the seeded supramolecular (co)-polymers were fitted using online amylofit software (http://www.amylofit.ch.cam.ac.uk) developed by Knowles and co-workers.[6] For an initial analysis, the half time of each reaction was extracted as the time point at which the degree of polymerization is half-way in between the initial baseline and final plateau values. The half time versus initial monomer concentration (m_0) was fitted by a power function

$$\text{half-time } (t_{1/2}) = A\, m_0^{\gamma} \quad \text{(S2)}$$

yielding a scaling exponent γ = -1.2 (Supplementary Figure 11a), -3.0 (Figure 5c), -2.0 (Supplementary Figure 18a), and -3.46 (Figure 7d) for homo-seeding, shear-induced supramolecular polymerization (60 RPM), 600 RPM and hetero seeding respectively. Lateral we have obtained the reaction orders form by the equations.

$$n_1 = -2\gamma \text{ (Primary nucleation reaction order)} \quad \text{(S3)}$$

$$n_2 = -(2\gamma+1) \text{ (Secondary nucleation reaction order)} \quad \text{(S4)}$$

In the case of homo-seeding experiments, the primary nucleation reaction order we obtained n_1 = 2.4, lateral we attempted to fit the normalization aggregation data with globally using set of models and it produce to best fit to the data is primary nucleation-elongation model. The fit shown in Supplementary Figure 11(b-e) is for $k_n = 1.20 \times 10^{-5}$, $k_e = 3.5 \times 10^6$. For homo seeding experiment, as fibre number concentration (P_0) is a required parameter, but experimentally it is very difficult to measure the numbers of fiber used as a seed solution.[5] Instead, the values for P_0 was chosen as arbitrarily (two or three orders of magnitudes smaller than initial aggregate concentration (M_0). So that, in homo-seeding seed-induced nucleation-elongation model fitting, P_0 was set as 10^{-9} M ($M_0 = 12.5 \times 10^{-6}$).

For shear-induced supramolecular polymerization (60 RPM) experiment as we mentioned above the scaling exponent (γ) value is -3.0 and we considered n_1 = 6 and n_2= 5, and we obtained the best fit to data from globally using set models is dominated secondary elongation models. The fit shown in Figure 5c is for $k_n k_+ = 1.20 \times 10^5$, $k_e k_+ = 3.5 \times 10^{12}$.

In the experiment involving stir-induced supramolecular polymerization at 600 RPM, as previously indicated, the scaling exponent (γ) is -2.0. With n_1 = 4 and n_2 = 3, we successfully fitted both the unseeded nucleation-elongation model (Supplementary Figure 18) and the unseeded secondary nucleation-elongation model (Supplementary Figure 19). The fitting in Supplementary Figure 18 corresponds to $k_n k_+ = 1.0 \times 10^7$. In Supplementary Figure 19, the fitting is for $k_n k_+ = 1.0 \times 10^{12}$, $k_e k_+ = 1 \times 10^{16}$.

For hetero-seeding experiments the scaling exponent is (γ) is -3.46 and we considered n_1 = 7 and n_2 = 6. From the various global set models, the best fit we obtained seed induced secondary nucleation-elongation models. The fit shown in Figure 7e is for $k_n k_+ = 1.20 \times 10^7$, $k_e k_+ = 3.5 \times 10^{16}$. The fibril number concentration (P_0) we considered was 10^{-9} M, whereas the seed concentration (M_0) is 25×10^{-6} M.

Mathematical Model: The mathematical models used to fit the experimental kinetics data were the analytical solutions of the seed-induced various models derived by Knowles and co-workers.[6] For instance, seed-induced nucleation-elongation, seed-induced secondary nucleation, Un-seeded secondary nucleation models were used in this present manuscript, the mathematical models are described as follows:

Nucleation-elongation Model:

$$\frac{M}{m_t} = 1 - \frac{m_0}{m_{tot}} \left(\frac{1}{\mu} cosh \left(sqrt \left(\frac{n_1}{2} \mu \lambda t + v \right) \right) \right)^{\frac{2}{n_1}} \tag{S5}$$

$$\lambda = sqrt \left(2k_+ k_n m_0^{n_1} \right) \tag{S6}$$

$$\mu = sqrt \left(\frac{k_+ n_1}{k_n m_0^{n_1}} P_0 \right) \tag{S7}$$

$$\alpha = sqrt \left(2k_+ k_n m_0^{n_1} \right) \tag{S8}$$

$$v = log \left(\alpha + \mu \right) \tag{S9}$$

3.3 Secondary nucleation:

$$\frac{M}{M_\infty} = 1 - \left(1 - \frac{M_0}{M_\infty} \right) e^{-k_\infty t} \left(\frac{B_- + C_+ e^{kt}}{B_+ + C_+ e^{kt}} \times \frac{B_+ + C_+}{B_- + C_+} \right)^{\frac{k_\infty}{k k'_\infty}} \tag{S10}$$

$$k = sqrt \left(2m_0 k_+ \frac{m_0^{n_2} k_2}{1 + m_0^{n_2} / k_m} \right) \tag{S11}$$

$$\lambda = sqrt \left(2k_+ k_n m_0^{n_1} \right) \tag{S12}$$

$$C_\pm = \frac{k_+ P_0}{k} \pm \frac{k_+ M_0}{2(m_0 k_+ - k_{off})} \pm \frac{\lambda^2}{2k^2} \tag{S13}$$

$$k_\infty = ksqrt \left(\frac{2}{n_2(n_2+1)} + \frac{2\lambda^2}{n_1 k^2} + \frac{2M_0}{n_2 m_0} + \left(\frac{2k_+ P_0}{k} \right)^2 \right) \tag{S14}$$

$$k'_{\infty} = sqrt\left(k_{\infty}^{2} - 2C_{+}C_{-}k^{2}\right) \quad \text{(S15)}$$

$$B_{\pm} = \frac{k_{\infty} + k'_{\infty}}{2k} \quad \text{(S16)}$$

where the mass at long times, M_{∞}, is just the total **2EH-PDI** mass concentration, m_{tot}. This solution is more accurate than the ones involving fragmentation, i.e. very close to the numerical integration of the differential equations, however it only applies for negligible off rates $k_{off} < k_{+}m_{0}$.

In the unseeded case this depends only on the combined rate constants $k_{+}k_{n}$ and $k_{+}k_{2}$, not k_{+}, k_{n} and k_{2} individually.

Parameters involved in above equations:

P = Fiber number concentration (number concentration of supramolecular polymer)

M_0 = Fiber mass concentration (mass concentration of supramolecular polymer)

m_0 = Monomer concentration

k_n = Primary nucleation rate constant

k_2 = Secondary nucleation rate constant

k_+ = Elongation rate constant

n_1 = reaction order of primary nucleation

n_2 = reaction order of secondary nucleation

2. Supplementary Figures

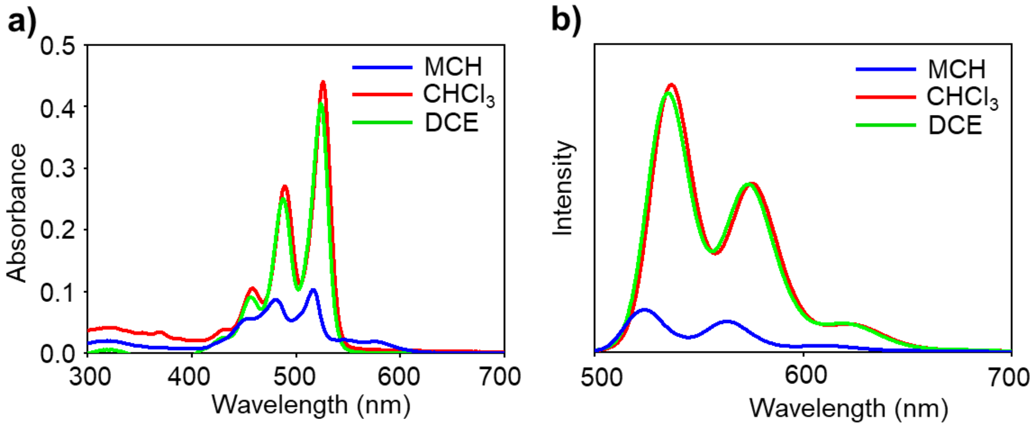

Supplementary Figure 1. Electronic (a) absorption and (b) fluorescence spectra of **2EH-PDI** (50 µM) in $CHCl_3$ (red), dichloroethane (DCE) (green), and methylcyclohexane (MCH) (Blue) (l = 1 mm, λ_{ex} = 460 nm). The fluorescence intensity was corrected according to the differences in the absorbance.

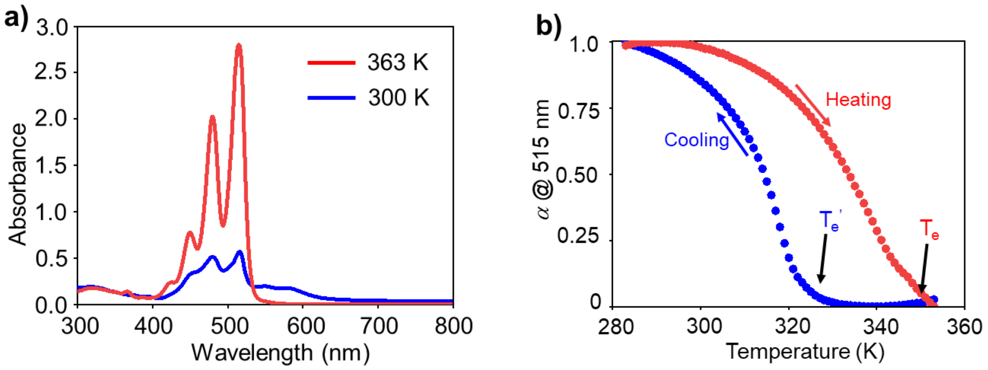

Supplementary Figure 2. (a) UV-vis spectra of **2EH-PDI** in MCH solvent at 363 K (red) and 300 K (blue) (c = 50 μM, l = 10 mm). (b) Plot of the degree of the polymerization (α) of **2EH-PDI** in MCH monitored at 515 nm vs temperature at a cooling rate of 1 K/min. (blue) and heating rate of 1 K/min. (red) (c = 50 μM).

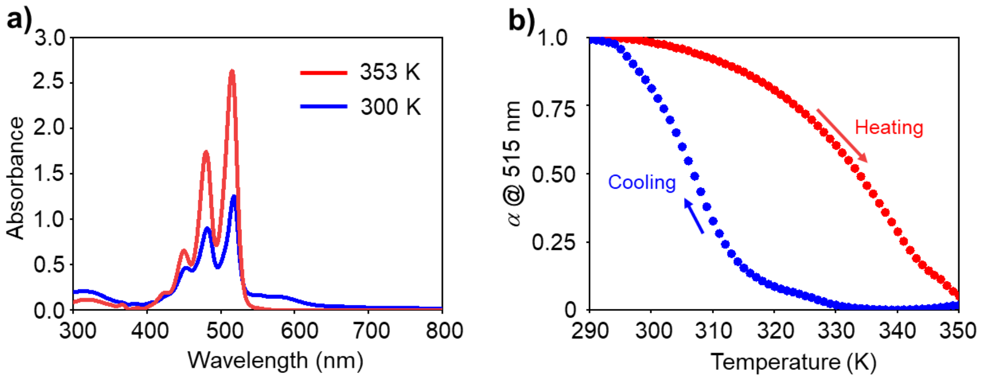

Supplementary Figure 3. (a) UV-vis spectra of **2EH-PDI** in 3% DCE in MCH solvent at 353 K (red) and 300 K (blue) (c = 50 μM, l = 10 mm). (b) Plot of the degree of the polymerization (α) of **2EH-PDI** in 3% DCE in MCH monitored at 515 nm vs temperature at a cooling rate of 1 K/min. (blue) and heating rate of 1 K/min. (red) (c = 50 μM).

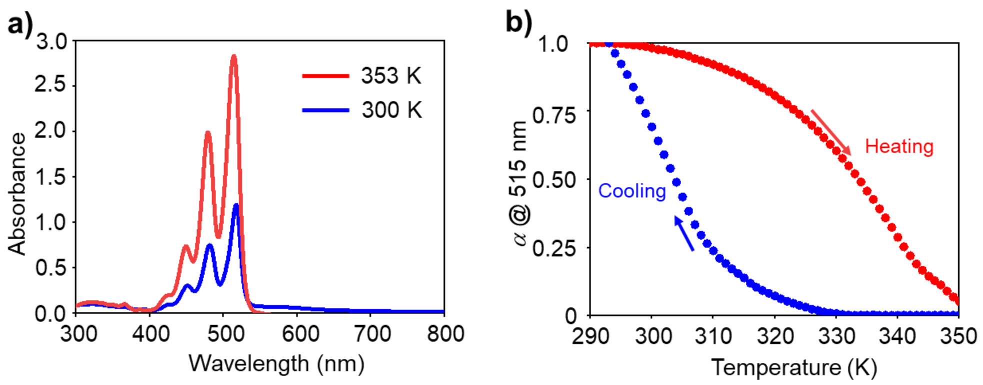

Supplementary Figure 4. (a) UV-vis spectra of **2EH-PDI** in 5% DCE in MCH solvent at 353 K (red) and 300 K (blue) (c = 50 μM, l = 10 mm). (b) Plot of the degree of the polymerization (α) of **2EH-PDI** in 5% DCE in MCH monitored at 515 nm vs temperature at a cooling rate of 1 K/min. (blue) and heating rate of 1 K/min. (red) (c = 50 μM).

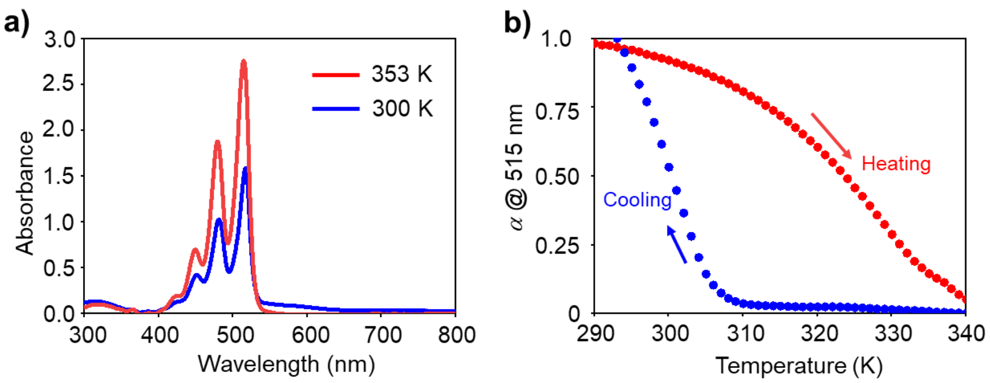

Supplementary Figure 5. (a) UV-vis spectra of **2EH-PDI** in 7% DCE in MCH solvent at 353 K (red) and 300 K (blue) (c = 50 μM, l = 10 mm). (b) Plot of the degree of the polymerization (α) of **2EH-PDI** in 7% DCE in MCH monitored at 515 nm vs temperature at a cooling rate of 1 K/min. (blue) and heating rate of 1 K/min. (red) (c = 50 μM).

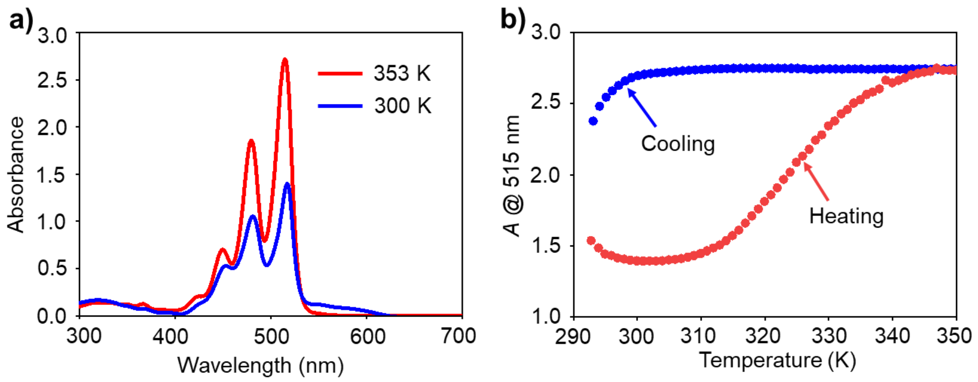

Supplementary Figure 6. (a) UV-vis spectra of **2EH-PDI** in 10% DCE in MCH (MCH*) solvent at 353 K (red) and 300 K (blue) (c = 50 μM, l = 10 mm). (b) Plot of absorbance (λ = 515 nm) vs temperature of **2EH-PDI** in 10% DCE in MCH (MCH*) at a cooling rate of 1 K/min. (blue) and heating rate of 1 K/min. (red) (c = 50 μM).

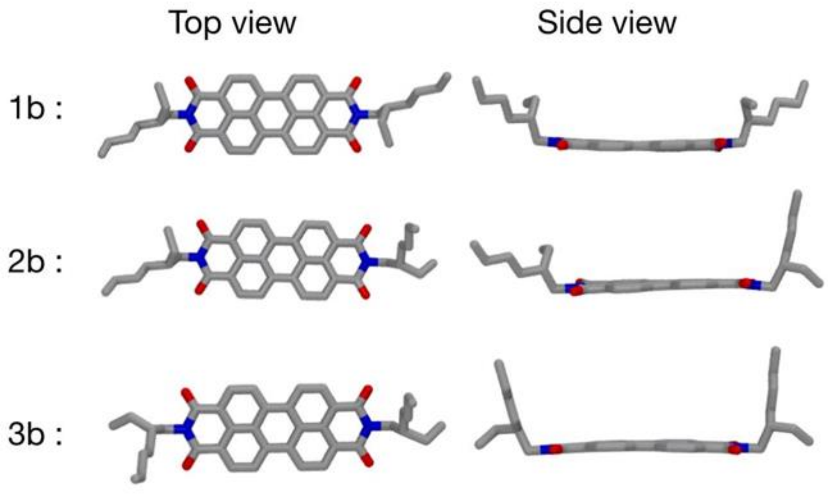

Supplementary Figure 7. Three (1b, 2b, 3b) most stable boat-type conformations of **2EH-PDI** molecule.

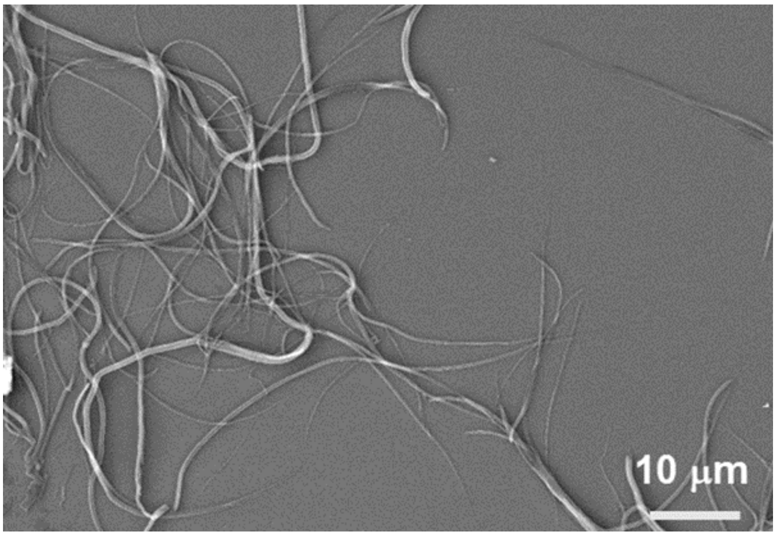

Supplementary Figure 8. FE-SEM image obtained by spin coating the solution of **2EH-PDI** solution in MCH* (c = 50 μM) on a silicon substrate. This image is representative of two experiments.

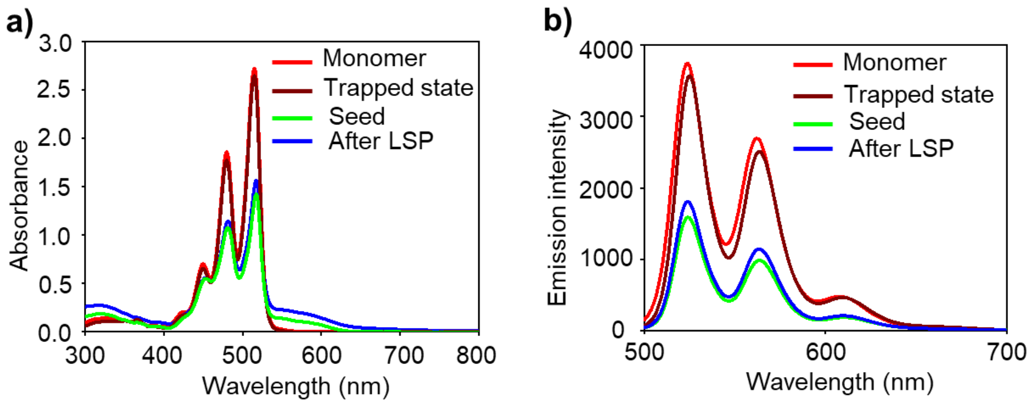

Supplementary Figure 9. (a) UV-vis spectra and (b) fluorescence spectra of **2EH-PDI** monomer (red), trapped state (wine), seed solution (green) and after performing LSP (blue) in MCH* ($c = 50$ µM, $l = 10$ mm, $\lambda_{ex} = 460$ nm at 303K). The fluorescence intensity was corrected according to the differences in the absorbance.

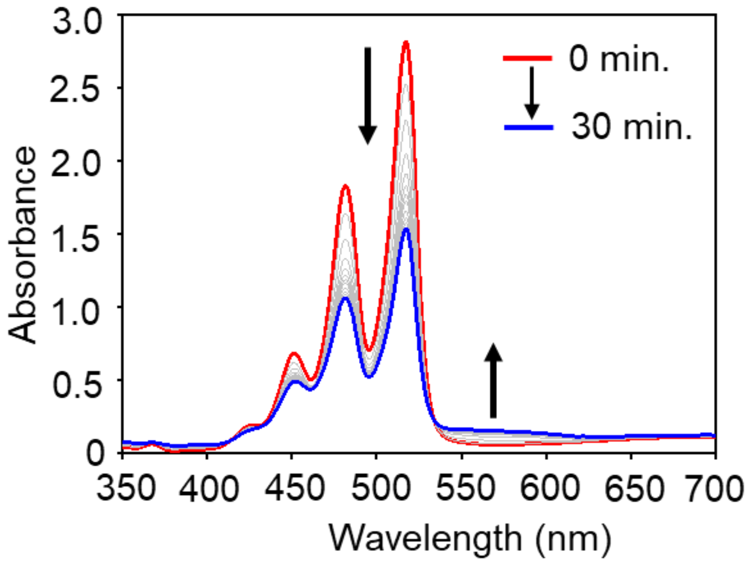

Supplementary Figure 10. Time dependent absorption spectral changes of **2EH-PDI** after addition of 5 mol% prefabricated **2EH-PDI** aggregates (*c* = 50 μM) after sonicating 1 hour at 303 K temperature. (*c* = 50 μM, *l* = 10 mm, Solvent: MCH*).

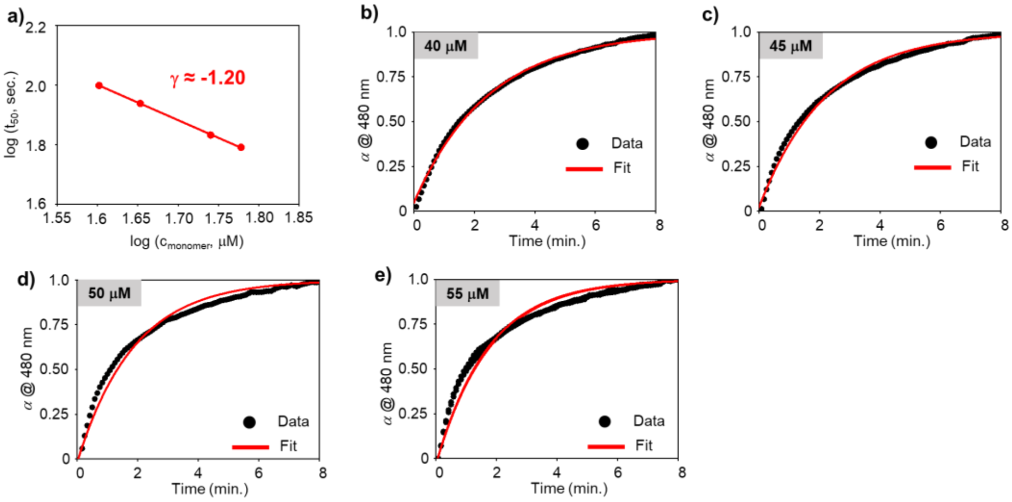

Supplementary Figure 11. (a) Log-log plot of rate of homo-seeded supramolecular polymerization with the various monomer concentrations of **2EH-PDI**, which shows a linear trend with a slope of -1.20 ± 0.09 suggesting a seeded chain-growth supramolecular polymerization process. (b-e) Fitting of kinetic data obtained via homo seeding into seed-induced nucleation-elongation model by using online software http://www.amylofit.ch.cam.ac.uk at constant seed concentration ([**2EH-PDI Seed**] = 12.5 µM) and variable dormant monomer concentration of **2EH-PDI**: b) 40 µM, c) 45 µM, d) 50 µM and e)55 µM (*α* @ 480= degree of supramolecular polymerization monitored at 480 nm, solvent: MCH*, *l* = 10 mm at 303K).

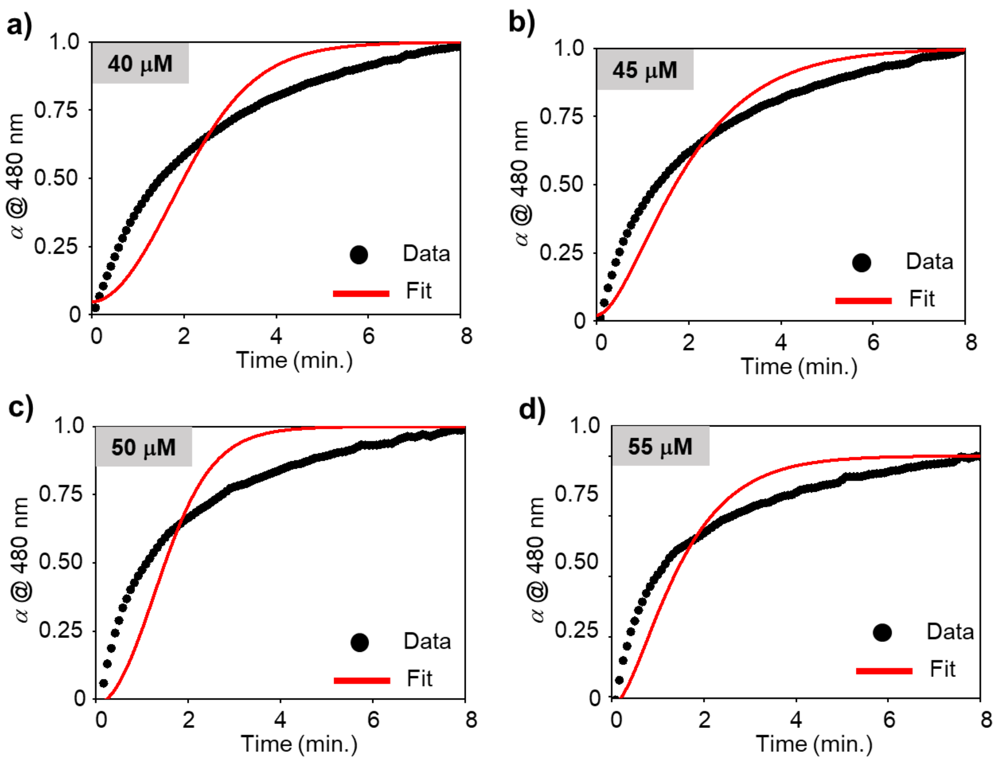

Supplementary Figure 12. (a-d) Fitting of kinetic data obtained via homo seeding into seed-induced secondary nucleation-elongation model by using online software http://www.amylofit.ch.cam.ac.uk at constant seed concentration ([**2EH-PDI Seed**] = 12.5 µM) and variable dormant monomer concentrations of **2EH-PDI**: a) 40 µM, b) 45 µM, c) 50 µM and d) 55 µM (*α* @ 480 = degree of supramolecular polymerization monitored at 480 nm, solvent: MCH*, *l* = 10 mm at 303K).

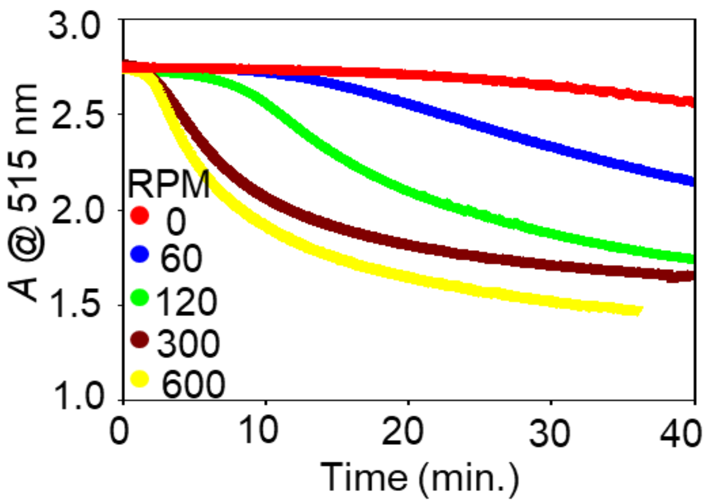

Supplementary Figure 13. Time-dependent variation in the absorbance of **2EH-PDI** dormant monomers at 515 nm under stirring at various RPM. (*c* = 50 μM, solvent: MCH* at 303K).

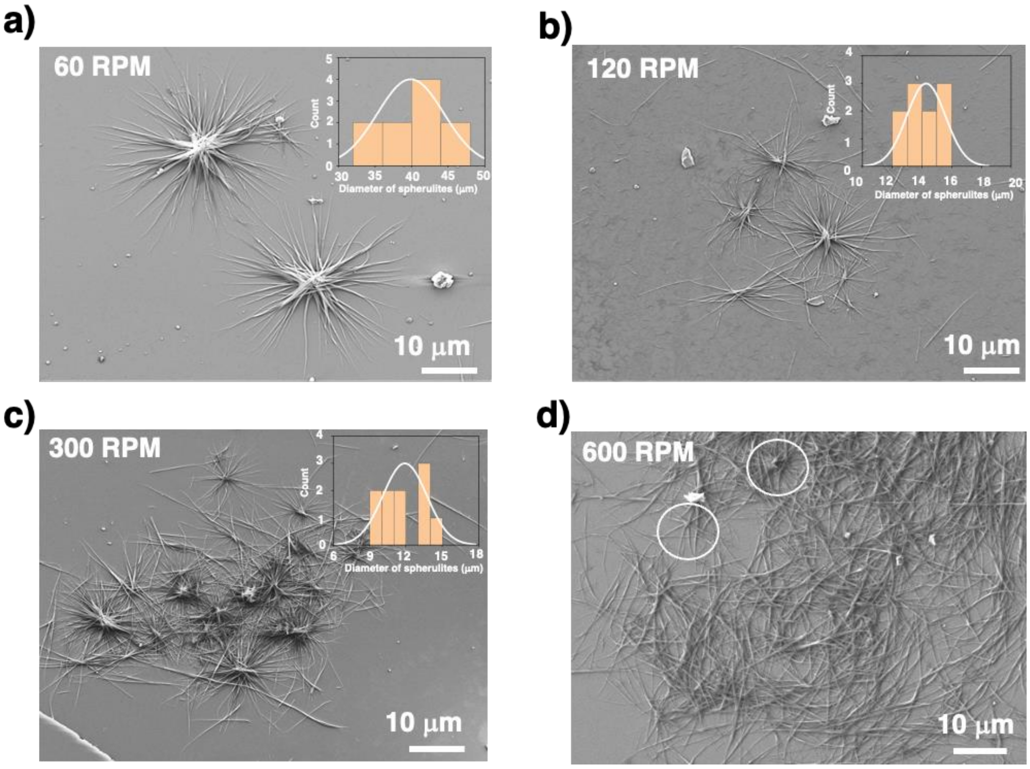

Supplementary Figure 14. FE-SEM images of **2EH-PDI** obtained after stirring the dormant monomers with various RPM (a) 60 RPM, (b) 120 RPM, (c) 300 RPM and (d) 600 RPM in MCH*. Inset: FE-SEM based histogram of the contour diameter of randomly selected 10 spherulites formed via 60 RPM (a), 120 RPM (b) and 300 RPM (c). The white circles in (d) represent the spherulite structures. (c = 50 μM, solvent: MCH* at 303K). All these images are representative of three experiments.

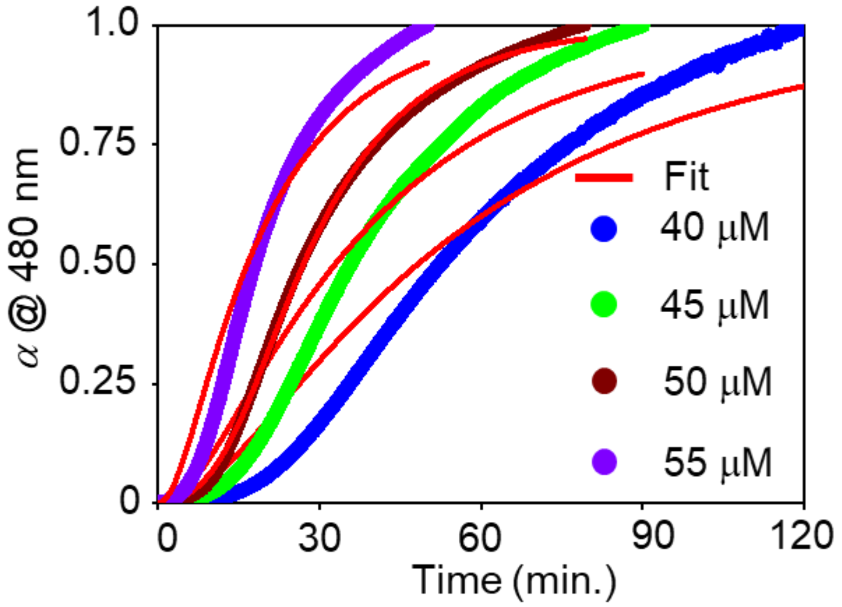

Supplementary Figure 15. Fitting of shear-induced kinetic data (stirring the solutions at 60 RPM) into unseeded nucleation-elongation model by using online software http://www.amylofit.ch.cam.ac.uk at various dormant monomer concentrations of **2EH-PDI** such as 40 μM, 45 μM, 50 μM and 55 μM. (α@ 480= degree of supramolecular polymerization monitored at 480 nm, solvent: MCH*, l = 10 mm at 303K).

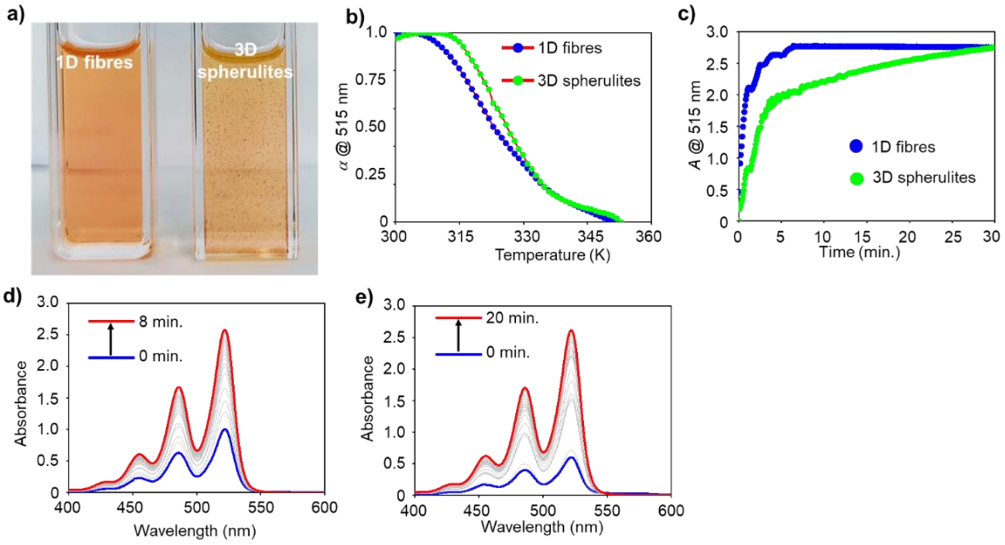

Supplementary Figure 16. (a) Photographs of solutions containing 1D fibres (left) and 3D spherulites (right) of **2EH-PDI**. (b) Melting analysis of 1D fibres (blue) and 3D spherulites (green) of **2EH-PDI** monitored at 515 nm (heating rate 1K/min.). (c) Time-dependent supramolecular depolymerization of 1D fibres and 3D spherulites of **2EH-PDI** after adding 70% DCE in MCH, monitored at 515 nm. (d) and (e) Time-dependent absorption spectra of dried **2EH-PDI** 1D fibres solution (d) and dried 3D spherulites solution (e) in 10 mm cell after adding 70% DCE in MCH. (c = 50 μM, *l* = 10 mm, Solvent: MCH*).

1D fibers were synthesized using a homo-seeding approach, incorporating 5% of sonicated pre-aggregated seeds into **2EH-PDI** dormant monomers. Simultaneously, 3D spherulites were synthesized by stirring **2EH-PDI** dormant monomers under 60 RPM for 1 hour. Subsequently, the solvent was removed and dried. To study the chemical stability of these 1D fibers and 3D spherulites, we have added 70% DCE in MCH to result a final concentration of 50 μM and recoded their UV-Vis absorption spectra at different time intervals at 303 K (Supplementary Figure 16c-e).

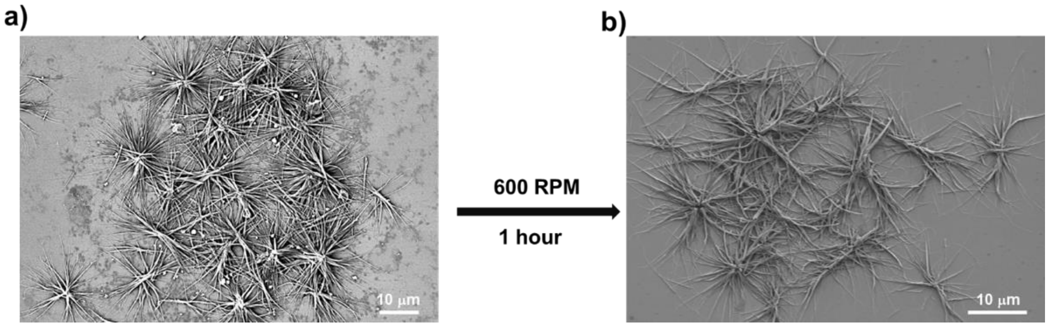

Supplementary Figure 17. (a) FE-SEM image of spherulites obtained from **2EH-PDI** at 60 RPM condition. (b) FE-SEM image of spherulites of **2EH-PDI** after stirring at 600 RPM for 1 hour. (solvent: MCH*, c = 50 μM, at 303K). The image in **a** is representative of three experiments, and the image in **b** is representative of one experiment.

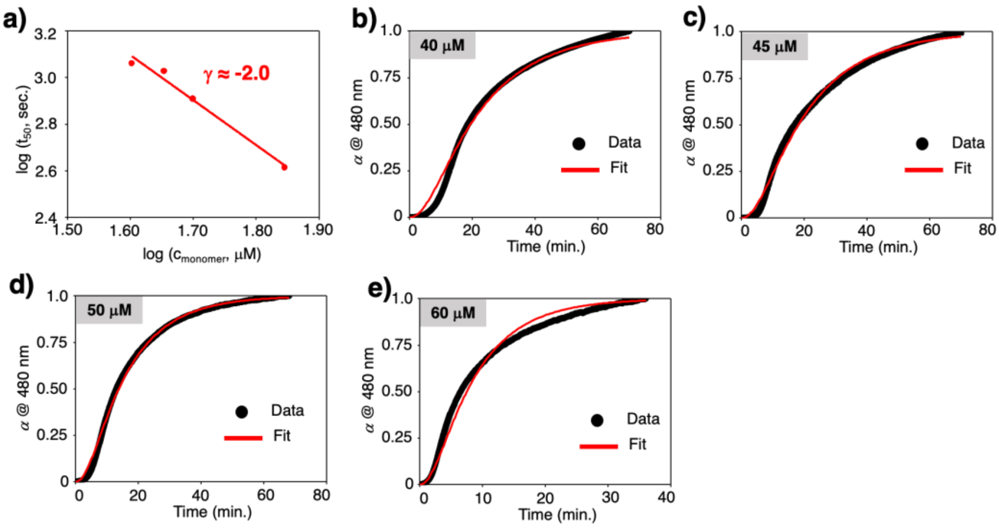

Supplementary Figure 18. (a) log-log plot of the half-times of stir-induced (600 RPM) supramolecular polymerization versus the original concentration of **2EH-PDI**. Symbols represent the experimental data; solid line is a power law fit. This fit shows a linear trend with a slope of -2.00 ± 0.374 referred to as the exponent coefficient (γ), indicating a monomer dependent supramolecular polymerization process. (b-e) Fitting of shear-induced kinetics at 600 RPM conditions into unseeded primary nucleation-elongation model by using online software http://www.amylofit.ch.cam.ac.uk at various dormant monomer concentration: b) 40 µM, c) 45 µM, d) 50 µM and e) 60 µM (*α* @ 480= degree of supramolecular polymerization monitored at 480 nm, solvent: MCH*, *l* = 10 mm at 303 K).

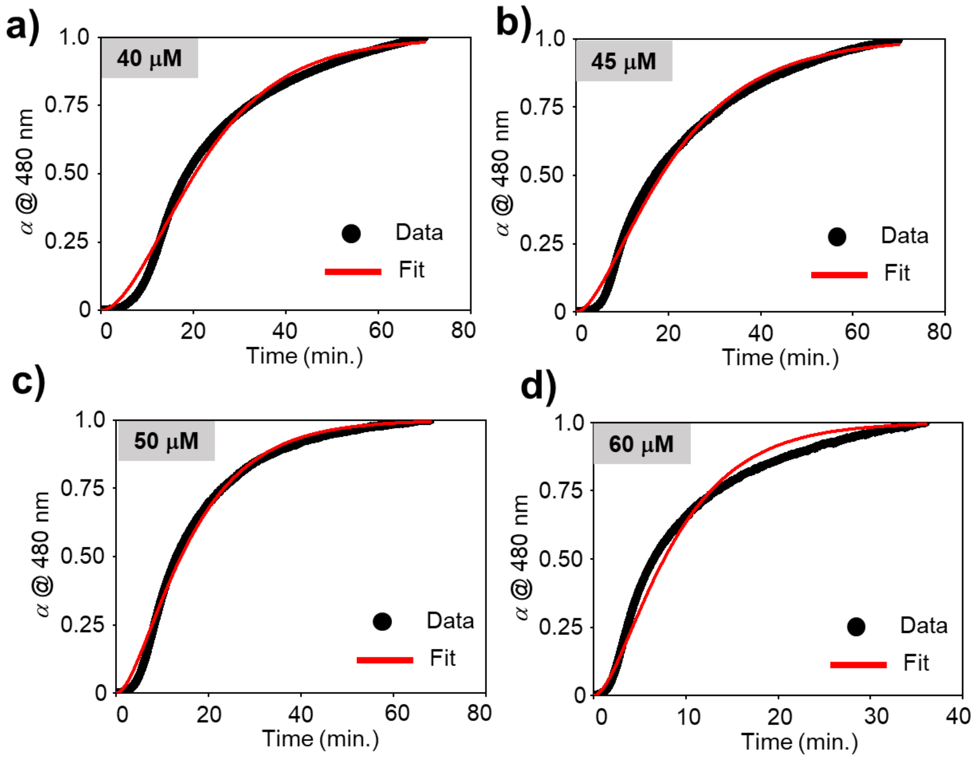

Supplementary Figure 19. (a-d) Fitting of shear-induced kinetics at 600 RPM conditions into unseeded secondary nucleation-elongation model by using online software http://www.amylofit.ch.cam.ac.uk at various dormant monomer concentrations of **2EH-PDI**: (a) 40 μM, (b) 45 μM, (c) 50 μM and (d) 60 μM (α @ 480 = degree of supramolecular polymerization monitored at 480 nm, solvent: MCH*, l = 10 mm at 303 K).

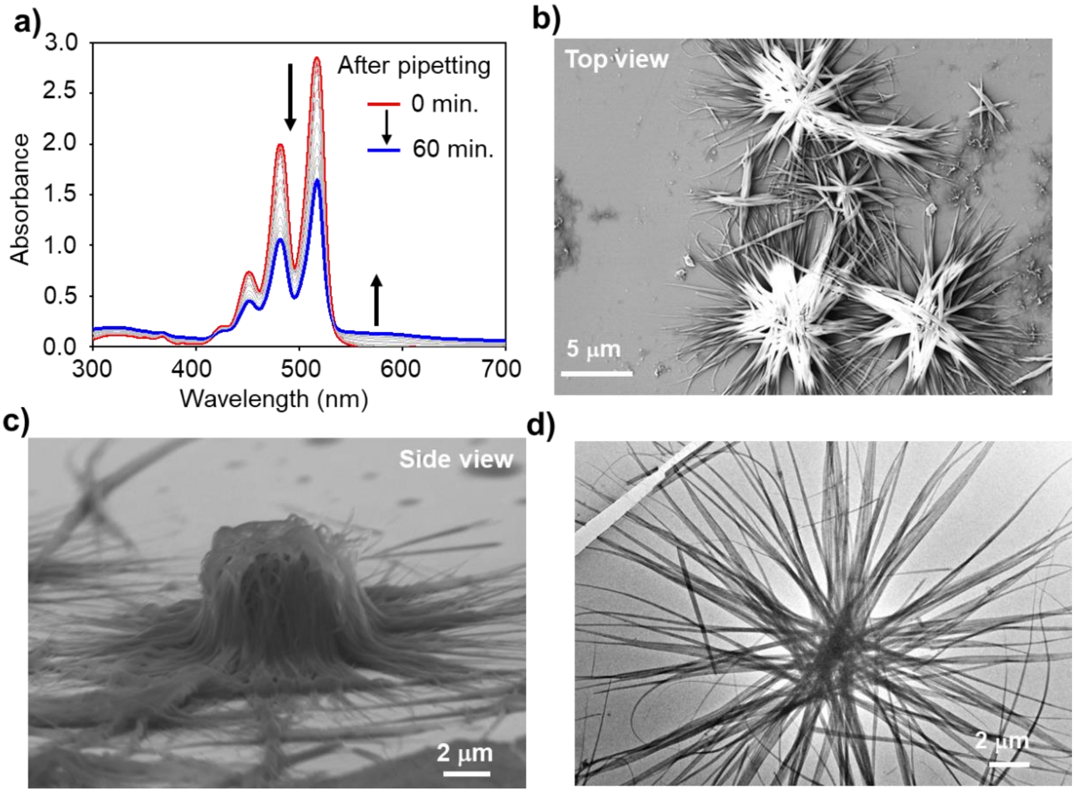

Supplementary Figure 20. (a) Time dependent absorption spectral changes of **2EH-PDI** dormant monomer solution after repetitive pipetting. (b) Top view FE-SEM image, (c) side view FE-SEM image (tilted angle, 70º) and (d) TEM image of **2EH-PDI** recorded after one hour of applying repetitive pipetting (c = 50 μM, l = 10 mm, Solvent: MCH* at 303K). The images in **b** and **c** are representative of three experiments, and the image in **d** is representative of two experiments.

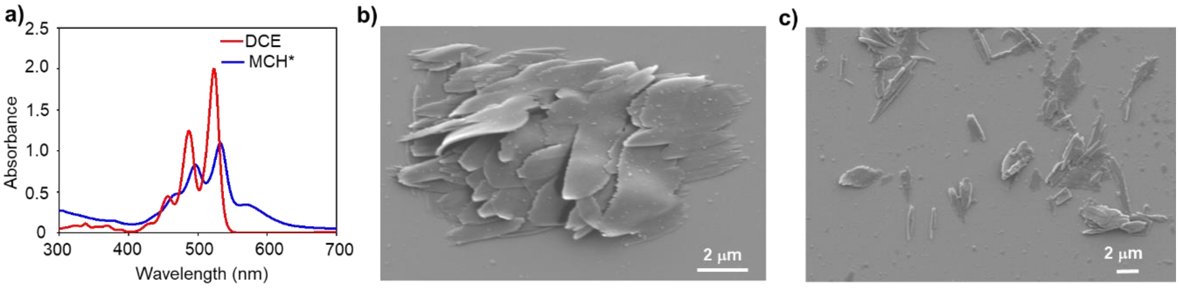

Supplementary Figure 21. (a) Electronic UV-vis absorption spectra of **PE-PDI** in DCE (red) and MCH* (blue). FE-SEM images of **PE-PDI** (b) before and (c) after sonication for 1 hour (c = 25 μM, l = 10 mm Solvent: MCH* at 303K). The images in **b** and **c** are representative of two experiments.

The UV-vis spectra suggest that **PE-PDI** (25 μM) in DCE exhibits a well-resolved absorption spectrum with vibrational features, indicating its monomeric form at 30 °C. However, in MCH*, the spectra show lower absorbance, and the emergence of a new red-shifted band centered at 575 nm, suggests the self-assembly of **PE-PDI** (Supplementary Figure 21a). FE-SEM studies of **PE-PDI** in MCH* reveal that the self-assembled aggregates of **PE-PDI** have 2D platelets like morphology (Supplementary Figure 21b).

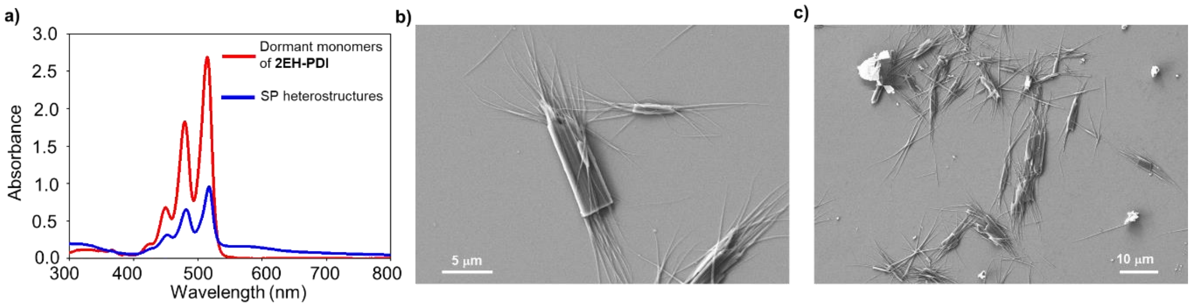

Supplementary Figure 22. (a) UV-vis spectra of **2EH-PDI** after the addition of 50 mol% prefabricated **PE-PDI** aggregates after sonicating 1 hour at 303 K temperature. (b) and (c) FE-SEM images of synthesized SP heterostructures via hetero seeding approach by adding 50 mol% **PE-PDI** seeds to **2EH-PDI** dormant monomers in MCH*. (c = 50 μM, l = 10 mm Solvent: MCH* at 303K). The images in **b** and **c** are representative of four experiments.

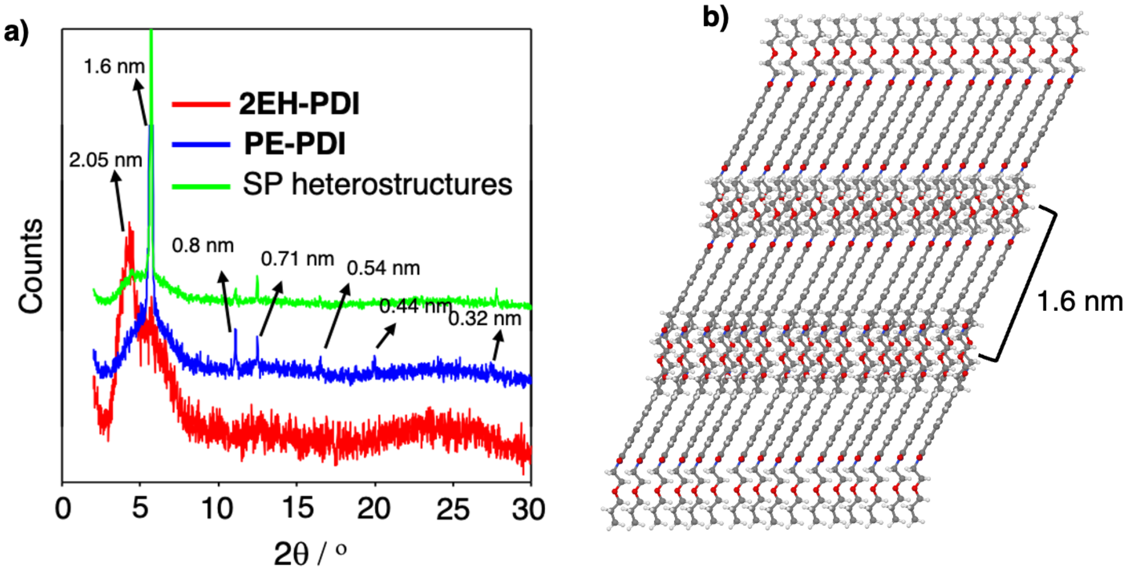

Supplementary Figure 23. (a) Thin film XRD pattern of **2EH-PDI** (red), **PE-PDI** (blue) and SP heterostructures (green) obtained via hetero seeding approach by adding 50 mol% **PE-PDI** seeds to **2EH-PDI** dormant monomers (50 μM) in MCH*. (b) Schematic illustration of proposed molecular packing of **PE-PDI** molecules in the 2D platelets.

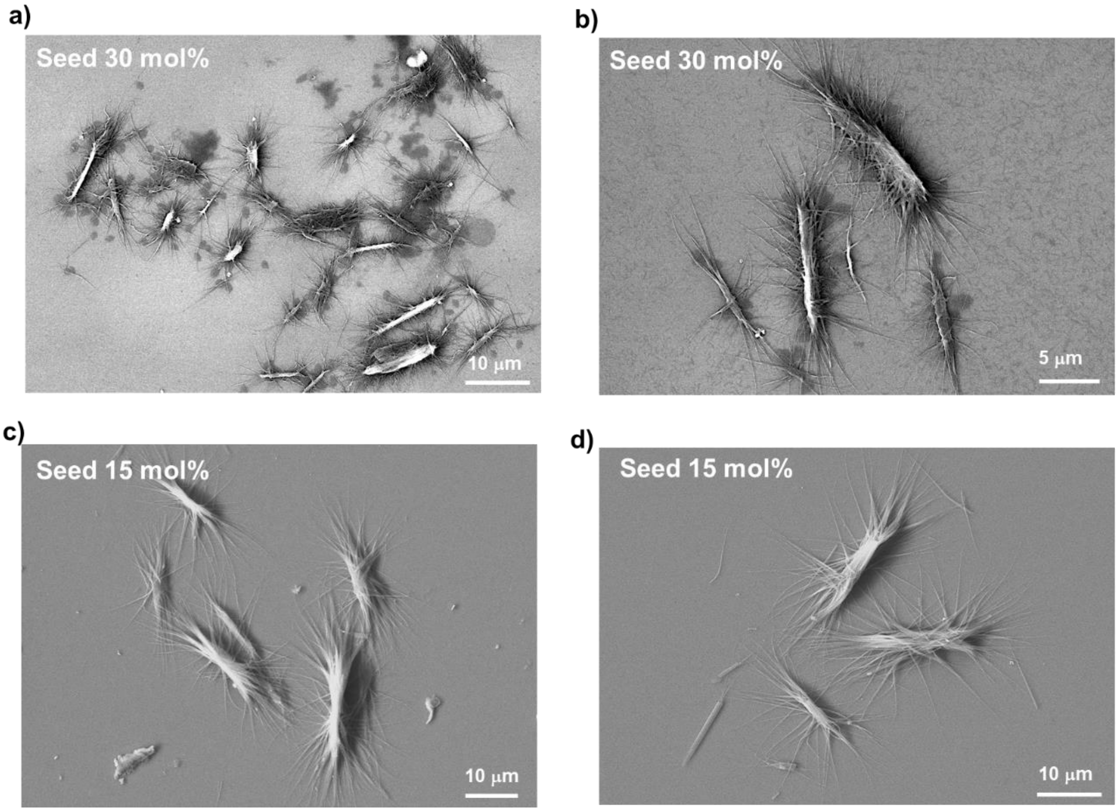

Supplementary Figure 24. FE-SEM images of synthesized SP heterostructures via hetero seeding approach by adding (a) and (b) 30 mol% **PE-PDI** seeds, (c) and (d) 15 mol% **PE-PDI** seeds to **2EH-PDI** dormant monomers in MCH*. All these images are representative of two experiments.

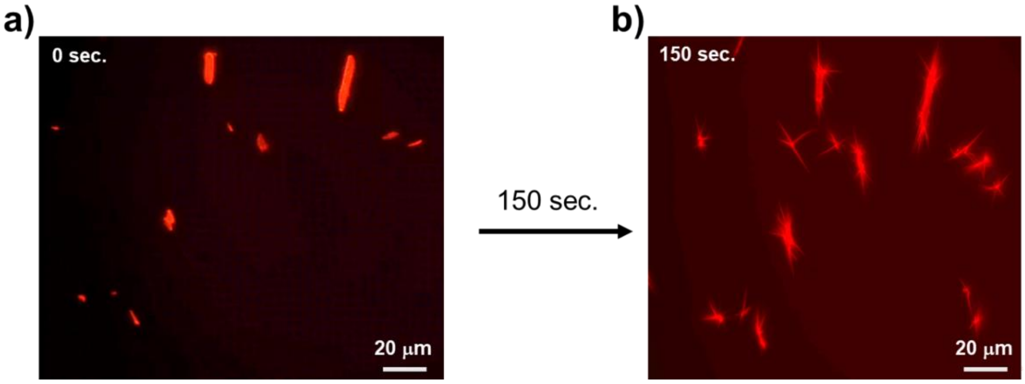

Supplementary Figure 25. Fluorescence microscopy images showing time-dependent growth of SP heterostructures after mixing the 50 mol% of **PE-PDI** seeds to the **2EH-PDI** (50 μM) dormant monomers in MCH* (a) at 0 seconds and (b) after 150 seconds.

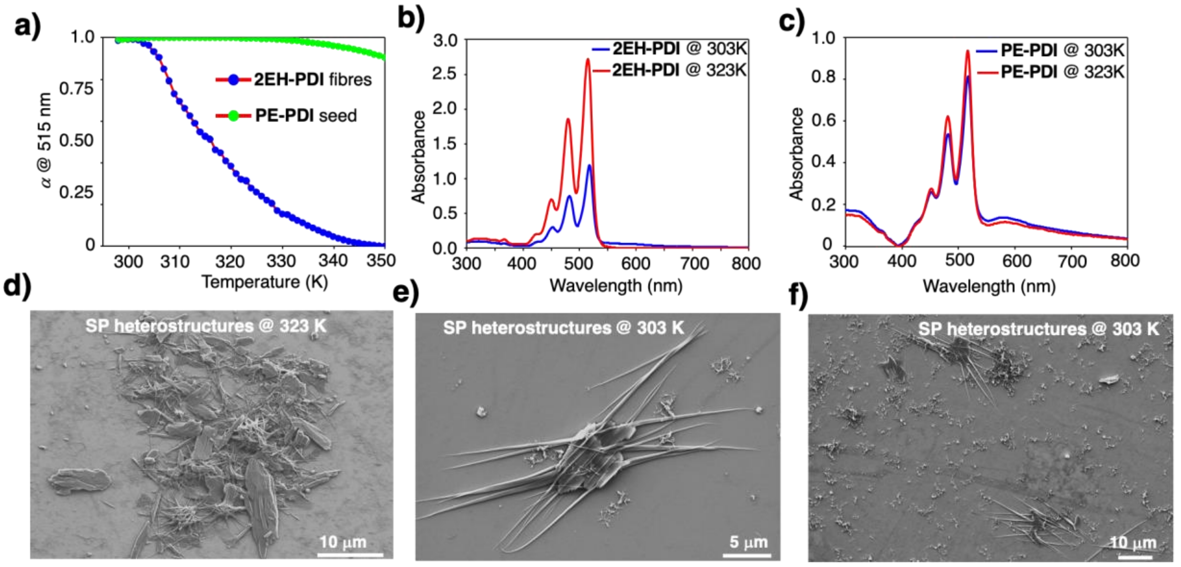

Supplementary Figure 26. (a) Melting analysis of **2EH-PDI** 1D fibers (50 μM, MCH*) and **PE-PDI** seed particles prepared by sonicating for 1 hour (25 μM, MCH*). (b) UV-vis spectra of **2EH-PDI** 1D fibres (50 μM, MCH*) at 303 K (blue) and 323 K (red). (c) UV-vis spectra of **PE-PDI** seed particles (25 μM, MCH*) at 303 K (blue) and 323 (red). (d) The FE-SEM image obtained by spin coating the solution containing SP heterostructures, which was heated at 323 K for 15 minutes. (e) and (f) FE-SEM images showing regeneration of SP heterostructures formed after cooling down the solution from 323 K to 303 and waiting for 1 hour. The images in **d**-**f** are representative of one experiment.

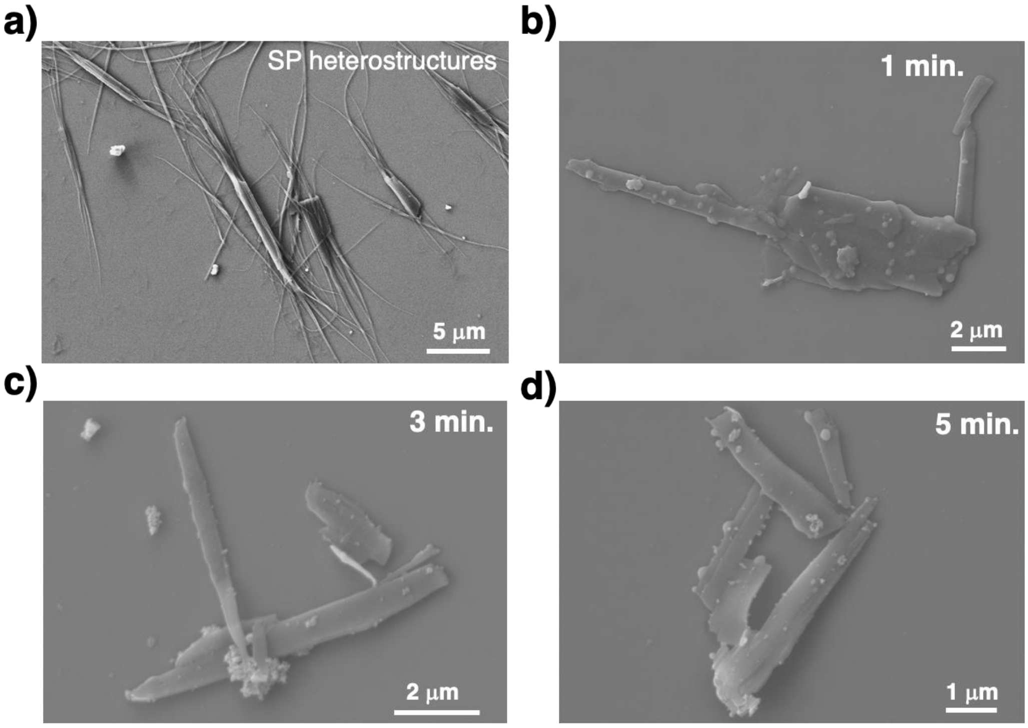

Supplementary Figure 27. (a) FE-SEM image of obtained SP heterostructures via hetero seeding approach (50 mol% of **PE-PDI** seed was added to the dormant monomers of **2EH-PDI**). FE-SEM images (b), (c) and (d) depict the removal of **2EH-PDI** fibers from SP heterostructures after dipping in MCH* at 323 K for various time intervals. The image in **a** is derived from four independent experiments and the images **b-d** are representative of one experiment.

3. Supplementary tables

Supplementary Table 1: Mean squared error (MSE) of homo-seeding experiments with varying the monomer concentration obtained after fitting the seeded kinetics with seed-induced primary nucleation-elongation model (Supplementary Figure 11 b-e) and seed-induced secondary nucleation-elongation model (Supplementary Figure 12 a-d).

Concentration of **2EH-PDI** Dormant monomer (μM)	Primary nucleation-elongation model	Secondary nucleation-elongation model
40	0.00015028	0.0061619
45	0.00033087	0.003574
50	0.000813	0.0093474
55	0.0009747	0.0057764

Supplementary Table 2: Mean squared error (MSE) of stir induced experiments (60 RPM) with varying the monomer concentration obtained after fitting the stir-induced kinetics with unseeded primary nucleation elongation model (Supplementary Figure 15) and unseeded secondary nucleation-elongation model (Figure 5c).

Concentration of **2EH-PDI** Dormant monomer (μM)	Primary nucleation-elongation model	Secondary nucleation-elongation model
40	0.0061619	0.00015028
45	0.003574	0.00033087
50	0.0093474	0.000813
55	0.0057764	0.0012747

Supplementary Table 3: Mean squared error (MSE) of stir induced experiments (600 RPM) with varying the monomer concentration obtained after fitting the stir-induced kinetics with unseeded primary nucleation elongation model (Supplementary Figure 18 b-e) and unseeded secondary nucleation-elongation model (Supplementary Figure 19a-d).

Concentration of **2EH-PDI** Dormant monomer (μM)	Primary nucleation-elongation model	Secondary nucleation-elongation model
40	0.000907	0.000877
45	0.0004318	0.00057263
50	0.0002777	0.0004938014
60	0.00136660	0.001655497

Supplementary Table 4: Mean squared error (MSE) of hetero-seeding experiments with varying the monomer concentration obtained after fitting the seed-induced secondary nucleation-elongation model (Figure 7e).

Concentration of **2EH-PDI** Dormant monomer (μM)	Secondary nucleation-elongation model
40	0.000640
45	0.00015
50	0.000176
62	0.000426

4. Supplementary References

(1) M. J. Frisch, G. W. Trucks, H. B. Schlegel, G. E. Scuseria, M. A. Robb, J. R. Cheeseman, G. Scalmani, V. Barone, G. A. Petersson, H. Nakatsuji, X. Li, M. Caricato, A. V. Marenich, J. Bloino, B. G. Janesko, R. Gomperts, B. Mennucci, H. P. Hratchian, J. V. Ortiz, A. F. Izmaylov, J. L. Sonnenberg, D. Williams-Young, F. Ding, F. Lipparini, F. Egidi, J. Goings, B. Peng, A. Petrone, T. Henderson, D. Ranasinghe, V. G. Zakrzewski, J. Gao, N. Rega, G. Zheng, W. Liang, M. Hada, M. Ehara, K. Toyota, R. Fukuda, J. Hasegawa, M. Ishida, T. Nakajima, Y. Honda, O. Kitao, H. Nakai, T. Vreven, K. Throssell, J. A. Montgomery, Jr., J. E. Peralta, F. Ogliaro, M. J. Bearpark, J. J. Heyd, E. N. Brothers, K. N. Kudin, V. N. Staroverov, T. A. Keith, R. Kobayashi, J. Normand, K. Raghavachari, A. P. Rendell, J. C. Burant, S. S. Iyengar, J. Tomasi, M. Cossi, J. M. Millam, M. Klene, C. Adamo, R. Cammi, J. W. Ochterski, R. L. Martin, K. Morokuma, O. Farkas, J. B. Foresman, and D. J. Fox. Gaussian 16, Revision B.01, Gaussian, Inc., Wallingford CT.

(2) Stewart, J. J. P. Optimization of Parameters for Semiempirical Methods V: Modification of NDDO Approximations and Application to 70 Elements. *Journal of Molecular Modeling* **2007**, *13*, 1173–1213.

(3) Chai, J.-D.; Head-Gordon, M. Long-Range Corrected Hybrid Density Functionals with Damped Atom–Atom Dispersion Corrections. *Phys. Chem. Chem. Phys.* **2008**, *10*, 6615-6620.

(4) Woods, J. F.; Gallego, L.; Pfister, P.; Maaloum, M.; Vargas Jentzsch, A.; Rickhaus, M. Shape-Assisted Self-Assembly. *Nat. Commun.* **2022**, *13*, 1-8.

(5) Humphrey, W.; Dalke, A.; Schulten, K. VMD: Visual Molecular Dynamics. *Journal of Molecular Graphics* **1996**, *14*, 33–38.

(6) Meisl, G.; Kirkegaard, J. B.; Arosio, P.; Michaels, T. C. T.; Vendruscolo, M.; Dobson, C. M.; Linse, S.; Knowles, T. P. J. *Nat. Protoc.* **2016**, *11*, 252–272.
