# Supplementary material for: Noncovalent synthesis of homo and hetero-architectures of supramolecular polymers via secondary nucleation
Source: Nat Commun. 2024 Apr 30;15:3672. doi: 10.1038/s41467-024-47874-5 (PMC11063220; doi:10.1038/s41467-024-47874-5)
Supplement: Supplementary file 3 — Description of Additional Supplementary Files [file 41467_2024_47874_MOESM3_ESM.pdf]

Description of Additional Supplementary Files

File Name: Supplementary Movie 1
Description: Growth of dormant monomers of 2EH-PDI having 50 mol% of PE-PDI seeds in MCH* in rectangular long capillary cells via hetero seeding. This video is captured using Olympus IX83 inverted fluorescence microscope under bright-field mode.

File Name: Supplementary Movie 2
Description: Growth of dormant monomers of 2EH-PDI having 50 mol% of PE-PDI seeds in MCH* in rectangular long capillary cells via hetero seeding. This video is captured using Olympus IX83 inverted fluorescence microscope under fluorescence mode.
